# RSO-YOLO: A Real-Time Detector for Small and Occluded Objects in Autonomous Driving Scenarios

**DOI:** 10.3390/s25216703

**Published:** 2025-11-02

**Authors:** Quanxiang Wang, Zhaofa Zhou, Zhili Zhang

**Affiliations:** School of Missile Engineering, Rocket Force University of Engineering, Xi’an 710025, China; wqx15609278062@163.com (Q.W.); zzl202@hhu.edu.cn (Z.Z.)

**Keywords:** autonomous driving, object detection, occluded objects, small objects

## Abstract

In autonomous driving, detecting small and occluded objects remains a substantial challenge due to the complexity of real-world environments. To address this, we propose RSO-YOLO, an enhanced model based on YOLOv12. First, the bidirectional feature pyramid network (BiFPN) and space-to-depth convolution (SPD-Conv) replace the original neck network. This design efficiently integrates multi-scale features while preserving fine-grained information during downsampling, thereby improving both computational efficiency and detection performance. Additionally, a detection head for the shallower feature layer P2 is incorporated, further boosting the model’s capability to detect small objects. Second, we propose the feature enhancement and compensation module (FECM), which strengthens features in visible regions and compensates for missing semantic information in occluded areas. This module improves detection accuracy and robustness under occlusion. Finally, we propose a lightweight global cross-dimensional coordinate detection head (GCCHead), built upon the global cross-dimensional coordinate module (GCCM). By grouping and synergistically enhancing features, this module addresses the challenge of balancing computational efficiency with detection performance. Experimental results demonstrate that on the SODA10M, BDD100K, and FLIR ADAS datasets, RSO-YOLO achieves mAP@0.5 improvements of 8.0%, 10.7%, and 7.2%, respectively, compared to YOLOv12. Meanwhile, the number of parameters is reduced by 15.4%, and model complexity decreases by 20%. In summary, RSO-YOLO attains higher detection accuracy while reducing parameters and computational complexity, highlighting its strong potential for practical autonomous driving applications.

## 1. Introduction

The continuous advancement of artificial intelligence has driven remarkable progress in autonomous driving. By incorporating semi-automated and fully automated modes, autonomous vehicles can reduce the operational complexity associated with human driving. In complex traffic scenarios, cooperative driving strategies are employed to optimize traffic flow and alleviate congestion [1]. During operation, autonomous driving systems typically rely on object detection techniques to achieve visual perception. These systems then process and analyze environmental information in real time, providing critical support for vehicle control [2].

Recently, deep learning-based object detection methods have accomplished remarkable progress across various domains, demonstrating superior performance. However, in autonomous driving, these algorithms still face significant challenges in complex, real-world traffic scenarios. The primary bottlenecks can be summarized in three aspects. First, detection performance for small objects remains suboptimal. In urban road environments, distant objects such as pedestrians and vehicles often occupy fewer than 32 × 32 pixels in images, resulting in insufficient feature representation and making accurate detection and recognition difficult. Second, detection reliability is considerably undermined by occlusion. Frequent partial or complete occlusions in complex traffic conditions lead to substantial loss of visual information, increasing the difficulty of recognition and raising the risk of both false positives (FPs) and false negatives (FNs). Third, balancing detection accuracy with computational efficiency remains challenging. Autonomous driving systems are required to operate reliably at high frame rates, which imposes strict constraints on computational efficiency. Yet, existing object detection algorithms regularly struggle to sustain high accuracy while meeting these efficiency demands.

To address these challenges, we propose an innovative RSO-YOLO based on YOLOv12 [3]. This approach enhances detection robustness for small and occluded objects while achieving an optimal balance between computational efficiency and detection accuracy. The model improves semantic integrity through multi-scale feature fusion and compensation mechanisms while optimizing the detection head to reduce both parameter count and computational complexity. Experimental results demonstrate that RSO-YOLO delivers strong detection performance in complex traffic scenarios without compromising computational efficiency. The key innovations of this work are summarized as follows:

1. We introduce the bidirectional feature pyramid network (BiFPN) [4] and space-to-depth convolution (SPD-Conv) [5]. These components replace the original neck network. BiFPN enables efficient multi-scale feature fusion through weighted bidirectional cross-scale connections, while SPD-Conv mitigates semantic information loss by preserving and reorganizing features in the spatial dimension. Together, they improve both computational efficiency and detection performance. Additionally, a shallow P2 branch is incorporated into the detection head to improve fine-grained feature perception, which is critical for reliable small-object detection.
2. We propose the feature enhancement and compensation module (FECM). This module enhances discriminative features in unoccluded regions and compensates for semantic deficiencies in occluded areas, thereby boosting the model’s capability to detect occluded objects. It also addresses feature loss issues commonly encountered in occlusion scenarios.
3. We design a lightweight global cross-dimensional coordinate detection head (GCCHead), built upon the global cross-dimensional coordinate module (GCCM). This lightweight detection head groups and synergistically enhances features to reduce computational complexity while improving detection accuracy. Its efficiency and performance make it particularly appropriate for real-time autonomous driving systems.

## 2. Related Work

### 2.1. General Object Detection

Deep learning-based object detection algorithms have significantly enhanced the environmental perception capabilities of autonomous driving systems through their powerful feature learning and representation abilities. Current mainstream detection architectures can be divided into two types: two-stage detectors and single-stage detectors. Two-stage detectors, such as the SPP-Net [6] and the R-CNN [7] series, rely on region proposal mechanisms. Although they achieve high detection accuracy, they are hindered by substantial computational complexity and inference latency, which make it difficult to meet the real-time requirements of autonomous driving applications. In contrast, single-stage detectors, such as the SSD [8] and the YOLO [9] series, employ end-to-end architectures that offer a favorable balance between detection accuracy and computational efficiency, rendering them more suitable for real-time autonomous driving applications. Additionally, the DETR [10] series, based on the Transformer [11] architecture, exhibits unique strengths in modeling long-range dependencies. However, their high training costs and slow inference speeds limit their practicality in autonomous driving systems. Overall, the YOLO series effectively balances detection accuracy and inference speed, achieving high performance while maintaining processing rates exceeding 100 frames per second (FPS). Consequently, YOLO-based algorithms have become the mainstream approach for detecting objects in autonomous driving scenarios.

**Figure 1:**
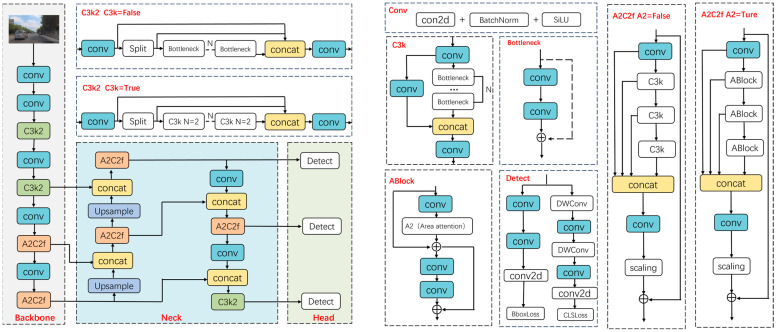
YOLOv12 network structure diagram. Arrows indicate data flow; colors distinguish module types; dashed boxes denote components; ⊕ denotes element-wise addition; the ellipsis “…” denotes repeated layers.

As a recent advancement in the YOLO series, YOLOv12 achieves significant developments in detection performance through the integration of a regional attention mechanism, with its architecture depicted in Figure 1. Its architecture contains three key components: the backbone, the neck, and the head. The backbone employs C3k2 and A2C2f to enable efficient feature extraction. The feature pyramid network (FPN) and path aggregation network (PAN) implement bottom-up multi-scale feature fusion and top-down semantic and fine-grained feature interaction, respectively. Features at different scales are fused via concatenation operations, while the A2C2f and C3k2 modules enhance the complementarity between semantic and fine-grained details. The detection head comprises three decoupled sub-heads, each containing independent classification and regression branches, thereby enabling task decoupling that enhances both computational efficiency and detection accuracy.

### 2.2. Detection of Small and Occluded Objects in Autonomous Driving Scenarios

#### 2.2.1. Small Object Detection

The detection of small objects in autonomous driving scenarios has remained a persistent research challenge. Li et al. [12] proposed Attention-YOLOv4, which enhances local feature extraction by integrating channel attention with residual blocks. However, this model is limited by high parameter complexity and slow inference speed. To address computational efficiency, Luo et al. [13] developed YOLOv8-ghost-EMA, which combines the Ghost and the EMA to improve feature representation while reducing parameter requirements. Similarly, Yang et al. [14] introduced MEL-YOLO, an extension of YOLOv5s. By incorporating multi-scale feature enhancement, decoupled detection heads, and a lightweight Ghost module, MEL-YOLO significantly improves small-object detection accuracy while maintaining lower model complexity. Yang et al. [15] designed LEAD-YOLO, which leverages a hierarchical feature fusion module (HFFM) to improve small object representation. However, its generalization ability remains to be validated. Wang et al. [16] proposed LKA-YOLO, which decouples large-kernel convolutions by introducing large kernel attention mechanisms. This structural optimization reduces computational complexity while improving detection performance for small objects.

#### 2.2.2. Occluded Object Detection

Significant research efforts have been directed toward enhancing YOLO-based models for occluded object detection in complex scenarios. Liu et al. [17] proposed RFCS-YOLO, which optimizes the YOLOv7 neck network by incorporating an efficient feature extraction module and a cross-scale fusion module, thereby improving traffic object detection. However, its high computational complexity reduces detection speed. Zhao et al. [18] proposed YOLO-BOS, which employs a Bi-level Routing Spatial Attention (BRSA) mechanism and a Shape-IOU loss function, significantly enhancing occlusion detection. Nonetheless, its increased computational cost restricts applicability in resource-limited environments. To address dense occlusion in traffic scenarios, Zheng et al. [19] developed YOLO-RC, integrating a feature extraction module with a multi-scale feature fusion strategy to achieve a lightweight design while maintaining occlusion detection performance. He et al. [20] introduced YOLO-OVD, which strengthens detection robustness in occluded scenes through attention mechanism optimization and refined loss functions. Wang et al. [21] introduced a coordinate attention network into VV-YOLO. By modeling both channel and spatial relationships within the neck network, the model can adaptively focus on key regions, thereby enhancing feature extraction performance in complex traffic scenes.

### 2.3. Model Lightweighting

Model lightweighting focuses on reducing network parameters and improving computational efficiency. In the general domain, architectures such as MobileNet [22] and EfficientNet [23] have been developed to achieve an optimal trade-off between speed and accuracy. Zhu et al. [24] developed MTNet, which lowers computational complexity by fusing convolutional and batch normalization layers and introducing lightweight feature fusion blocks to minimize redundant computations. This approach significantly reduces both computational and memory overhead while maintaining accuracy. Similarly, Chen et al. [25] introduced the MINIAOD algorithm, employing Ghost convolution to build a lightweight feature extraction network. By leveraging a feature map redundancy retention strategy, MINIAOD preserves discriminative capability while reducing computational overhead, ensuring robust feature representation.

In autonomous driving, numerous studies have refined YOLO-based models to attain an effective compromise between accuracy and speed. Gu et al. [26] proposed YOLO-ML, which integrates MobileNet v3 as the backbone of YOLOv4 and introduces a feature fusion redistribution mechanism along with a multi-scale feature interaction strategy, achieving strong trade-offs between accuracy and speed. Bie et al. [27] developed YOLOv5n-L, which significantly reduces parameter count by replacing standard convolutional structures with depthwise separable convolutions and C3Ghost modules, while maintaining real-time detection performance. Chen et al. [28] introduced PCPE-YOLO, which dynamically adjusts module structures and optimizes feature processing to substantially lower model parameters and computational overhead, making it well-suited for edge deployment in diverse scenarios. Yuan et al. [29] proposed YOLOv5s-A2, which introduces a Path Fusion Module (PA) to enhance feature integration capabilities and designs an Attention Detection Head (ADH) to suppress feature interference.

In summary, while significant strides have been made in the three areas discussed above, most methods are optimized for only one or two challenges and lack algorithms capable of simultaneously addressing multiple objectives, including small object detection, occlusion handling, and lightweight design. In real-world autonomous driving scenarios, these challenges often occur concurrently, making it difficult for existing algorithms to satisfy real-time requirements while maintaining both high accuracy and robustness.

## 3. Methods

### 3.1. Design of RSO-YOLO Network Architecture

#### 3.1.1. Structural Composition of RSO-YOLO

**Figure 2:**
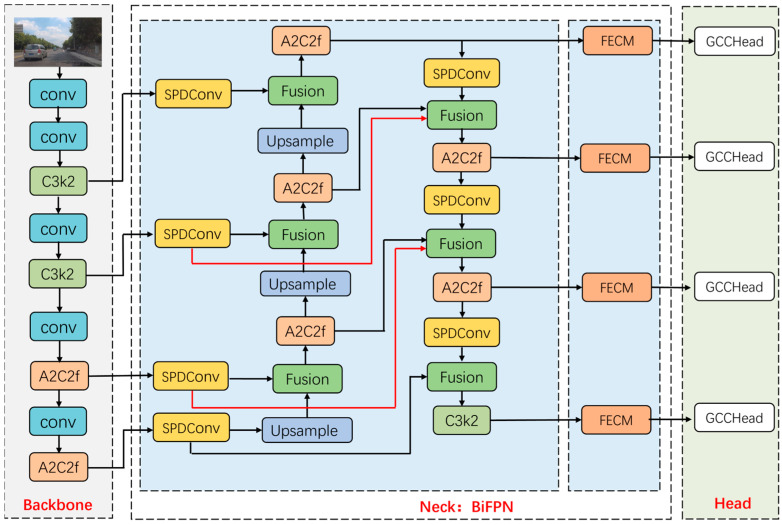
RSO-YOLO network structure diagram. Arrows indicate data flow; colors distinguish module types; dashed boxes denote components.

This paper proposes RSO-YOLO, an innovative model for detecting small and occluded objects in autonomous driving scenarios. The proposed model’s architecture, depicted in Figure 2, consists of three fundamental components: the Backbone, the BiFPN, and the Head.

The core improvements can be summarized as follows:

First, the BiFPN and SPD-Conv structures are introduced to replace the original neck network. The BiFPN enhances computational efficiency through effective bidirectional cross-scale feature fusion. The SPD-Conv replaces the traditional downsampling convolution in BiFPN by remapping spatial information to the channel dimension during sub-sampling, thereby preserving fine-grained details and improving feature representation for small objects. Additionally, a shallower P2 detection head is incorporated, leveraging high-resolution features for supervision to further enhance small object detection performance.

Next, the FECM is designed to enhance the discriminative power of critical regions by amplifying effective features in unoccluded areas using multi-scale branch-cooperative channels and spatial attention. Building upon this, FECM transfers high-confidence cues from unoccluded regions to occluded positions, reconstructs missing semantic information, and employs exponential amplification to strengthen weak target responses. This design significantly improves the model’s robustness in occlusion scenarios.

Finally, we propose a lightweight GCCHead. This detector leverages the GCCM introduced in this study. By grouping features, GCCM models global context along both height and width dimensions while adaptively enhancing feature responses in key regions. This approach improves detection accuracy while effectively controlling parameter count and computational complexity, enabling the model to achieve an optimum balance between performance and real-time inference requirements.

#### 3.1.2. Design Rationale

Rather than a mere combination of existing modules, the architecture of RSO-YOLO is designed around a cooperative interaction between spatial and channel features. Specifically, the integration of BiFPN and SPD-Conv establishes a spatial–channel feature enhancement mechanism, in which BiFPN strengthens semantic representations through bidirectional multi-scale fusion, while SPD-Conv preserves fine-grained spatial information by re-mapping it into the channel dimension during downsampling. This synergy ensures richer semantic consistency and better retention of discriminative details, which are critical for accurate small-object detection. Furthermore, the FECM and GCCHead modules enhance the model’s robustness and computational efficiency. FECM transfers high-confidence cues from visible to occluded regions, effectively compensating for missing semantic information. Meanwhile, GCCHead adopts a lightweight cross-dimensional coordination mechanism that improves detection accuracy while significantly reducing model parameters and computational cost. The proposed framework attains an optimal balance among feature completeness, robustness, and real-time performance, which represent fundamental requirements for achieving reliable perception in autonomous driving systems.

### 3.2. BiFPN and SPD-Conv

To tackle the problem of perceiving small objects in autonomous driving, this paper introduces the BiFPN and SPD-Conv. Together, these components enable efficient cross-scale feature fusion while maximizing the preservation of fine-grained information.

#### 3.2.1. BiFPN

**Figure 3:**
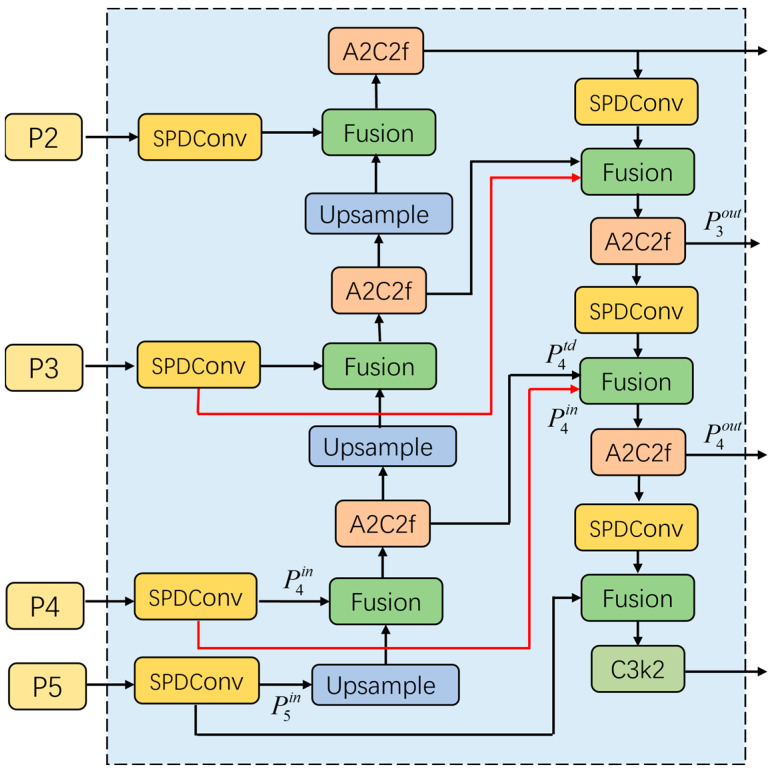
The structure of BiFPN. Arrows indicate data flow; colors distinguish module types; dashed box denotes components.

BiFPN is a bidirectional FPN with weighted feature redistribution that effectively integrates feature representations across different scales. Its structure is depicted in Figure 3. It takes a multi-scale feature list ${\vec{P}}^{in}=(P_{l_{1}}^{in},P_{l_{2}}^{in},\ldots )$ as input, where $P_{l_{i}}^{in}$ represents the features of the $l_{i}$ layer, BiFPN performs bidirectional fusion to generate a new list of multi-scale features as the ${\vec{P}}^{\mathrm{out}}=f({\vec{P}}^{\mathrm{in}})$ output.

BiFPN implements a weighted fusion mechanism to handle the uneven contributions from feature maps arising from heterogeneous input resolutions. This approach assigns a trainable weight coefficient to each input branch, moving beyond a simple uniform fusion. This mechanism allows the network to adaptively rebalance feature contributions based on their relative importance. We adopt the fast normalized fusion scheme in this work, which is shown in Equation (1):

(1) $$O=\sum_{i}\frac{w_{i}}{\in +\sum_{j}w_{j}}\cdot I_{i}$$

where $w_{i}$ represents the weight coefficient of the i-th input feature, and $I_{i}$ denotes the corresponding input feature map. To prevent numerical instability, a small constant ∈=0.0001 is added to the denominator. The normalized weights generated by this scheme are constrained within the range of 0 to 1 and do not require the use of a Softmax operation, thereby improving computational efficiency.

The BiFPN employed in this study integrates SPD-Conv subsampling, and a fast normalization fusion mechanism. The feature fusion at the fourth layer, as a specific instance, is mathematically formulated in Equations (2) and (3).

(2) $$P_{4}^{td}=Block\left(\frac{w_{1}\cdot P_{4}^{in}+w_{2}\cdot Resize\left(P_{5}^{in}\right)}{w_{1}+w_{2}+\in }\right)$$

(3) $$P_{4}^{\mathrm{out}}=Block\left(\frac{w_{3}\cdot P_{4}^{in}+w_{4}\cdot P_{4}^{td}+w_{5}\cdot Resize\left(P_{3}^{\mathrm{out}}\right)}{w_{3}+w_{4}+w_{5}+\in }\right)$$

where $P_{4}^{td}$ represents the intermediate transition feature of the P4 scale in the top-down path; $P_{4}^{\mathrm{out}}$ denotes the final output feature of P4 in the top-down path after bidirectional fusion; Block indicates convolutional processing unit, for which we substitute the standard convolution with the A2C2f module. Resize represent the upsampling and downsampling operations, respectively.

#### 3.2.2. SPD-Conv

**Figure 4:**
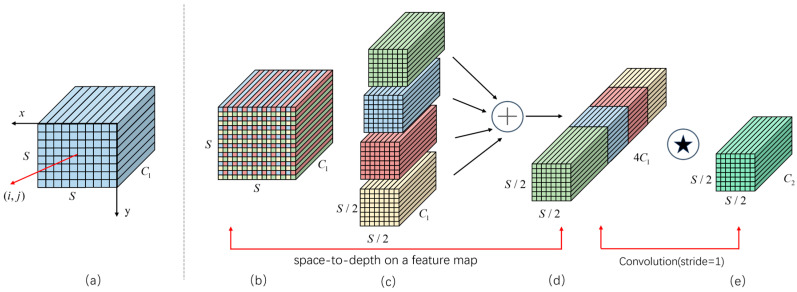
The structure of SPD-Conv: (**a**) input feature map; (**b**) spatial partition; (**c**) spatial-to-channel rearrangement; (**d**) channel compression; (**e**) output feature map. Arrows indicate data flow; colors distinguish module types; letters C and S denote the number of channels and the image size, respectively; ⊕ denotes concatenation of feature maps along the channel dimension; ⍟ denotes a convolution operation with a stride of 1.

Addressing the problem of fine-grained information loss inherent in traditional subsampling methods, SPD-Conv is introduced as a replacement for the standard convolution in BiFPN. This replacement maintains input feature details during downsampling, which boosts the model’s capacity for capturing discriminative features. The architecture of SPD-Conv integrates a space-to-depth transformation with a non-strided convolution. Its structure is depicted in Figure 4.

The SPD module partitions the input feature map into smaller patches based on a predefined downsampling ratio. It then remaps spatial information from the two-dimensional plane into the channel dimension through resampling. Subsequently, a 1 × 1 convolution layer processes the expanded channels and compresses them to achieve compact yet efficient feature representation. When the scale factor is set to 2, the SPD module increases the number of channels by a factor of four while reducing the spatial resolution by half, thereby enabling near-lossless reconstruction of the original content. This design effectively alleviates the degradation typically introduced by conventional convolution-based downsampling, allowing the network to retain fine-grained details of small objects and ultimately enhancing detection accuracy for small targets.

### 3.3. FECM

**Figure 5:**
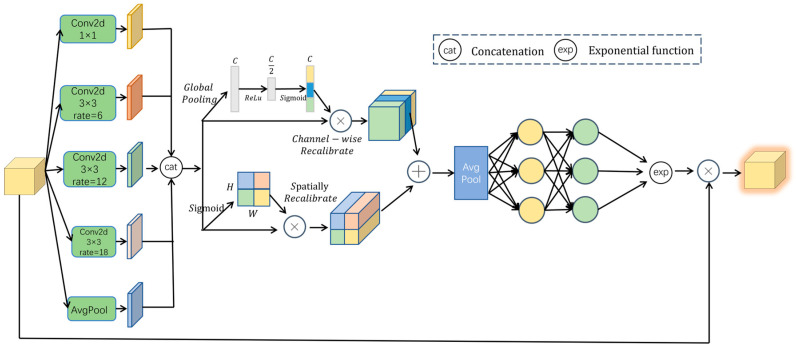
The structure of FECM. Arrows indicate data flow; colors distinguish module types; letters C, H, and W denote the number of channels, height, and width, respectively; ⊕ denotes element-wise addition; ⊗ denotes element-wise multiplication.

In autonomous driving scenarios, targets are frequently obscured, leading to a substantial decline in detection accuracy due to feature loss in traditional methods. To overcome this challenge, we propose the FECM. As illustrated in Figure 5, this module strengthens discriminative features in unoccluded regions while compensating for missing semantic information in occluded areas, thereby boosting the model’s strength under occlusion.

The FECM adopts a five-branch parallel architecture. Specifically, a 1 × 1 convolution branch preserves the original spatial resolution, while three 3 × 3 dilated convolution branches (with dilation rates of 6, 12, and 18) progressively enlarge the receptive field. In addition, global average pooling is applied to extract semantic information at the global level, providing contextual supplementation in cases where local textures are missing. The features from all parallel branches are integrated through channel-wise concatenation into a composite representation, which is subsequently refined through joint channel and spatial attention calibration. The two resulting attentional outputs are fused via additive combination, followed by a 1 × 1 convolution for dimensionality reduction and integration. This process enhances salient features in unoccluded regions, thereby improving the discriminative power of critical areas. To further strengthen information exchange between channels, a two-layer fully connected network is introduced. This mechanism transfers high-confidence cues from unoccluded regions to occluded positions, thereby reinforcing correlations between occluded and unoccluded targets and compensating for missing semantic details. Finally, an exponential transformation is applied to the enhanced features, extending the weight range from [0, 1] to [1, e]. This preserves relative ordering, reduces sensitivity to small-value perturbations, and improves robustness against positional deviations. The results of this transformation are then multiplied with the original features, significantly improving detection performance under occlusion.

### 3.4. GCCHead

**Figure 6:**
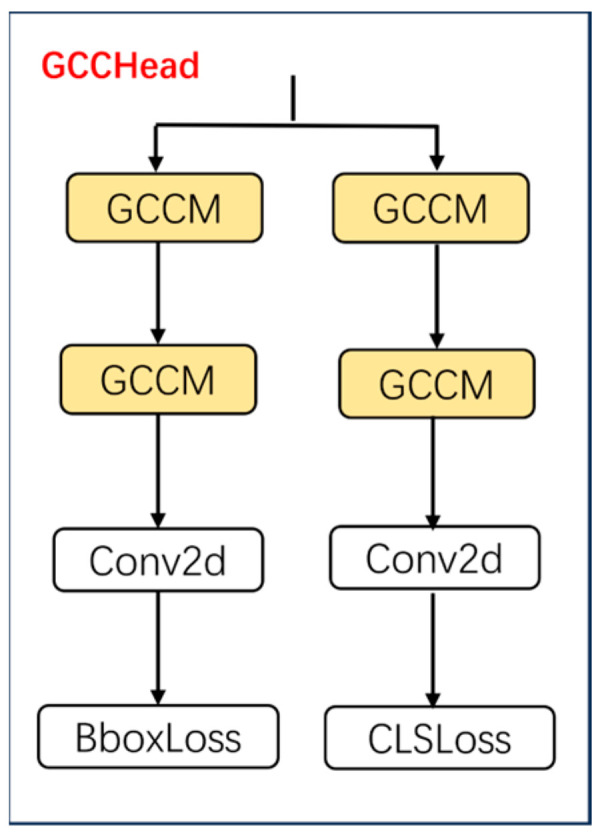
The structure of GCCHead.

To improve the detection head’s perceptive ability, a lightweight GCCHead is proposed, and its structure is shown in Figure 6. Unlike traditional YOLO detection heads that depend solely on standard convolutions, the GCCHead integrates multiple GCCM before the classification and regression branches. This design diminishes model complexity while strengthens its capacity to capture global contextual information. Consequently, the GCCHead maintains a lightweight structure while boosting the detection accuracy of the proposed model.

**Figure 7:**
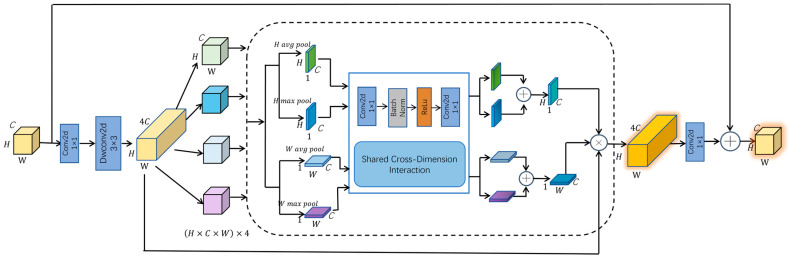
The structure of GCCM. Arrows indicate data flow; colors distinguish module types; letters C, H, and W denote the number of channels, height, and width, respectively; ⊕ denotes element-wise addition; ⊗ denotes element-wise multiplication.

Among these components, GCCM adopts grouped processing combined with a multi-dimensional attention mechanism, which significantly reduces computational complexity while boosting its capability to capture global multi-dimensional information, and its structure is shown in Figure 7. This design enables more effective feature representation.

The GCCM begins with a 1 × 1 convolution for channel expansion, enriching feature representations for subsequent processing. It then employs depthwise separable 3 × 3 convolutions to extract spatial information on a channel-by-channel basis, significantly reducing computational load and parameter count while maintaining effective receptive fields. Following channel compression and recovery via the shared cross-dimension interaction unit, the grouped features are aggregated, summed, and passed through a sigmoid function to generate horizontal and vertical attention maps. This process enables element-wise modulation of the expanded features for enhancement. A 1 × 1 convolution then reduces the channel count before the output is combined with the input through a residual path. By leveraging grouped processing, the GCCM effectively controls computational overhead, while global context modeling and multi-dimensional attention emphasize critical features and suppress irrelevant responses, thereby improving object detection performance.

## 4. Experiments

### 4.1. Datasets

#### 4.1.1. SODA10M Dataset

The SODA10M dataset [30] contains six object categories relevant to autonomous driving: tricycle, tram, car, cyclist, truck, and pedestrian. The images were collected across 32 cities, covering urban, highway, and rural environments under diverse weather conditions and times of day, thereby ensuring substantial environmental diversity and complexity. For this study, 10,000 labeled images were sampled and allocated to the test, validation, and training sets with a distribution of 20%, 20%, and 60%, respectively.

To further assess the model’s detection performance under occlusion conditions, the SODA10M test set was divided into occluded and non-occluded subsets. Since the SODA10M dataset does not provide explicit occlusion annotations, a heuristic criterion was adopted to identify occluded objects. Specifically, when the overlap between a target’s bounding box and those of other objects exceeded 20% of its area, the target was labeled as occluded; otherwise, it was labeled as non-occluded.

To evaluate the detection performance of RSO-YOLO across different object scales, a fine-grained assessment was conducted on the SODA10M test set. Following the official COCO evaluation protocol, all ground-truth objects were classified into three subsets based on their bounding box areas: small (area < 32^2^ pixels), medium (32^2^ ≤ area ≤ 96^2^ pixels), and large (area ≥ 96^2^ pixels).

#### 4.1.2. BDD100K Dataset

The BDD100K dataset [31] covers a wide range of driving environments under varying weather and lighting conditions (day, night, dusk, and dawn). For this study, 10,000 images were selected and categorized into four classes: car, person, traffic light, and traffic sign. The dataset was allocated to the test, validation, and training sets with a distribution of 20%, 20%, and 60%, respectively.

#### 4.1.3. FLIR ADAS Dataset

The FLIR ADAS dataset [32] includes a diverse range of scenes, comprising both thermal and visible-light images captured under various illumination and weather conditions. In this study, 5000 visible-light images were selected and annotated into three categories: pedestrian, bicycle, and car. The dataset was then divided into training, validation, and testing subsets in proportions of 60%, 20%, and 20%, respectively.

### 4.2. Experimental Environment and Parameters

We conducted all experiments on the VirtAI Cloud platform using a B1.medium instance equipped with 16 GB of system memory and a 12 GB GPU. The software environment was configured with Ubuntu 22.04, Python 3.10, and PyTorch 2.0.1 compiled with CUDA 11.8.

The training involved resizing all images to 640 × 640 pixels. We used the SGD optimizer with an initial learning rate of 0.01, a momentum of 0.937, and a weight decay of 0.0005. The learning rate was modulated by a cosine annealing schedule, which smoothly reduces the learning rate from the initial value to zero over the course of training, aiding convergence. The training spanned 300 epochs with a batch size of 16. This batch size was selected as the maximum feasible value given our GPU memory constraints, which helps stabilize the training process. The hyperparameter values, including the initial learning rate, scheduler, and optimizer settings, were primarily adopted from the official YOLOv12 configuration to ensure a fair comparison. Their effectiveness was further confirmed through initial validation experiments on the SODA10M validation set.

In addition, the overall loss function is divided into two types: regression and classification losses. The regression loss uses the CIoU localization loss to measure the geometric difference between predicted and ground-truth boxes. The classification loss includes the Dynamic Focal Loss for objectness prediction and the Meta-Contrastive Loss for category discrimination. Their weights are set to 0.8, 0.3, and 0.2, respectively, to balance the contribution of each component during training.

### 4.3. Evaluation Metrics

The model was evaluated across three dimensions: complexity, speed, and accuracy. Complexity was quantified using parameter count (Params) and GFLOPs. Speed was measured in FPS during inference. Accuracy was assessed using mean average precision (mAP), along with Precision (P) and Recall (R). The corresponding definitions are provided in Equations (4)–(7).

(4) $$P=\frac{TP}{TP+FP}$$

(5) $$R=\frac{TP}{TP+FN}$$

(6) $$AP=\int_{0}^{1}PdR$$

(7) $$mAP=\frac{1}{N}\sum_{i=1}^{N}AP_{i}\;$$

The mAP is defined as the macro-average of per-class average precision (AP) across N categories, providing a single metric that summarizes overall detection quality. For a given class, AP corresponds to the area under the precision–recall curve. Precision (P) denotes the proportion of predicted positive instances that are correct, while Recall (R) represents the proportion of actual positive instances that are successfully retrieved. True positives are correctly detected positive instances, FP are negative instances incorrectly predicted as positive, and FN are positive instances missed by the detector.

### 4.4. Ablation Experiment

#### 4.4.1. Ablation Experiments on the SODA10M Dataset

A systematic ablation study was conducted on the SODA10M dataset using the baseline model to validate the individual and collective contributions of the four enhancement strategies: BiFPN, P2-Detect, GCCHead, and FECM. The experimental results, detailed in Table 1, use a checkmark “√” to indicate the inclusion of a specific component.

In single-module experiments, each improvement contributed to performance gains. First, substituting the original neck with BiFPN enhanced the efficiency of multi-scale feature fusion. The introduced SPD-Conv preserved fine-grained information, yielding improvements of 0.7% in mAP@0.5 and 0.6% in mAP@0.5:0.95. Concurrently, a 35.4% reduction in computational complexity and a 15.4% decrease in parameter count were achieved, which collectively elevated the inference speed to 130 FPS. This demonstrates that the network not only reduced model complexity but also improved runtime efficiency while enhancing detection accuracy. Second, adding the P2 detection head strengthened high-resolution feature extraction for small objects, boosting mAP@0.5 by 4.5% and mAP@0.5:0.95 by 5.0%. However, because the P2 branch processes high-resolution features, GFLOPs increased accordingly. Third, replacing the baseline detection head with GCCHead reduced the number of parameters by 15.4%, while enhancing accuracy, demonstrating that GCCHead achieves a favorable trade-off between model compactness and detection accuracy. Finally, incorporating FECM into the baseline improved feature representation in unoccluded regions and compensated for semantic loss in occluded areas. This yielded an increase of 0.8% in both mAP metrics, with only a negligible growth in parameter count.

In the multi-module combination experiments, the proposed strategies exhibited strong complementarity. When BiFPN was combined with P2-Detect, the former accelerated multi-scale information exchange through efficient cross-layer fusion, while the latter introduced detection supervision on fine-grained low-level features to improve sensitivity to small objects. With parameters and computational cost remaining under control, this configuration sustained real-time inference and improved accuracy, yielding gains of 6.0% and 5.1% in mAP metrics over the baseline. Further replacing the original detection head with GCCHead achieves improvements of 7.0% and 6.0% in mAP metrics, while achieving a recall rate of 60.5% and reducing the parameter count by 19.2%. These results demonstrate that substituting standard convolutions with GCCM effectively reduces computational overhead while enhancing feature extraction. Finally, incorporating the FECM module further strengthened detection performance in occluded scenarios, raising the precision to 77.1%. This configuration also delivered improvements of 8.0% and 7.0% in mAP metrics, achieving the best results across all experimental settings.

**Figure 8:**
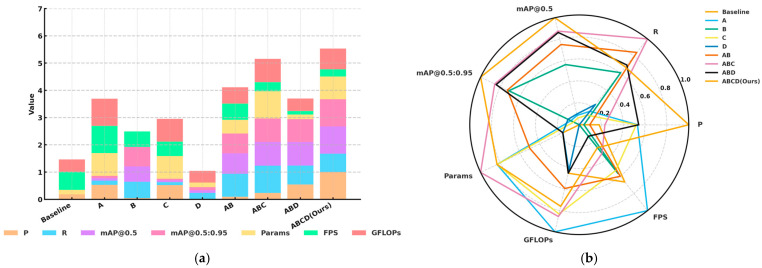
Normalized performance of ablation experiment on the SODA10M dataset: (**a**) normalized histogram of ablation experiments; (**b**) radar chart of ablation experiment performance.

In addition, Figure 8 (all data normalized, where smaller values indicate better performance for parameters and model size, and larger values indicate better performance for P, R, mAP metrics, and FPS) illustrates both the performance of individual indicators and the overall performance of RSO-YOLO. As shown in Figure 8a, models integrating strategy combinations (A, B, C, and D) consistently outperformed the baseline across key metrics. The radar chart in Figure 8b offers an intuitive overview, highlighting that the model ABCD (Ours) achieved the most balanced performance. Notably, it sustained strong results in critical indicators such as mAP@0.5, mAP@0.5:0.95, and FPS, while keeping parameter count and computational cost within practical limits.

Overall, ablation results verify the individual and synergistic contributions of the four improvement strategies to both computational efficiency and accuracy. When combined, these modules enabled RSO-YOLO to achieve optimal performance while preserving real-time capability, underscoring its potential for deployment in practical autonomous driving applications.

#### 4.4.2. Ablation Experiments on the BDD100K Dataset

To further assess the robustness and generalization of the proposed modules under varying training configurations, an additional ablation study was conducted on the BDD100K dataset. As shown in Table 2, each enhancement module consistently improved the model’s performance relative to the baseline. Incorporating the BiFPN module effectively strengthened multi-scale feature fusion, yielding increases of 0.8% in mAP@0.5 and 0.7% in mAP@0.5:0.95, while simultaneously reducing both the parameter count and computational complexity. The introduction of the P2 detection head produced the most significant single-module gain, improving mAP@0.5 by 4.6% and mAP@0.5:0.95 by 1.8%, which confirms its strong ability to enhance small object detection despite a moderate increase in GFLOPs. Replacing the standard detection head with GCCHead maintained accuracy while slightly lowering parameter count, achieving a balanced trade-off between detection precision and model compactness. The addition of the FECM module further refined semantic representation in occluded regions, offering steady improvements in both precision and recall. When multiple modules were combined, their complementary strengths became more apparent. The combination of BiFPN and P2 significantly improved mAP@0.5 by 6.1% compared with the baseline, while maintaining real-time inference capability. Substituting the detection head with GCCHead brought an additional 0.9% gain in mAP@0.5 and 0.4% in mAP@0.5:0.95, with negligible computational increase. Further integrating FECM enhanced detection robustness under complex and partially occluded conditions. The final configuration achieved 72.5% precision, 65.9% mAP@0.5, and 31.3% mAP@0.5:0.95, representing an optimal balance among accuracy, speed, and computational efficiency.

**Figure 9:**
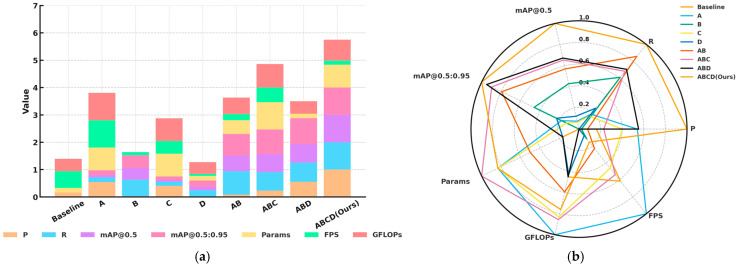
Normalized performance of ablation experiment on the BDD100K dataset: (**a**) normalized histogram of ablation experiments; (**b**) radar chart of ablation experiment performance.

Building on the previous analysis, Figure 9 further compares the normalized performance of models with different module combinations on the BDD100K dataset. All metrics were normalized, where smaller values indicate better performance for Params and GFLOPs, and larger values represent improvements in P, R, mAP, and FPS. As shown in Figure 9a, each enhancement module contributed positively to model performance, while their combinations produced cumulative benefits, reflecting the synergistic effects of multi-module integration. The radar plot in Figure 9b clearly demonstrates that the final configuration ABCD (Ours) achieved the most balanced results, maintaining high precision, recall, and inference speed while effectively controlling model size and computational complexity, thereby attaining an optimal balance among accuracy, efficiency, and robustness.

### 4.5. Comparative Experiments

To ensure a fair and consistent comparison, all compared models were trained and evaluated under the same experimental protocol. Specifically, the input size was fixed at 640 × 640, the training process lasted for 300 epochs, and the same optimizer configuration using SGD with a momentum of 0.937 and a weight decay of 0.0005 was applied to all models.

#### 4.5.1. Neck Network Comparative Experiment

The proposed BiFPN was benchmarked against the original YOLOv12 neck and the generalized feature pyramid network (GFPN) [33] on the SODA10M dataset to assess its efficacy, with findings summarized in Table 3. GFPN enhanced mAP metrics by 1.1% and 1.0% over the baseline but incurred an additional 0.4M parameters. In contrast, the BiFPN introduced in this study integrated SPD-Conv to maintain fine-grained details during multi-scale fusion, achieved improvements of 0.7% and 0.6% in mAP metrics, respectively, while reducing parameters by 0.4M. Although its accuracy is marginally lower than GFPN, BiFPN uses 0.8M fewer parameters, striking a superior balance between accuracy and complexity and confirming its practical utility.

#### 4.5.2. Performance Comparison Experiment of Occluded Object Detection Based on FECM

An evaluation was conducted on the occlusion subset of the SODA10M dataset to validate FECM’s efficacy in handling occlusion, with findings summarized in Table 4. Compared to the YOLOv12 baseline, FECM achieved improvements of 1.2% and 0.9% in the mAP metrics with a marginal parameter increase. These results indicate that the FECM module effectively compensates for occluded regions through context-aware feature enhancement, significantly boosting detection robustness.

#### 4.5.3. Detection Head Comparative Experiment

A comparative analysis on the SODA10M dataset evaluates the proposed GCCHead against the original YOLOv12 detection head and v10Detect, with findings summarized in Table 5. The GCCHead demonstrates a 0.4% gain in mAP@0.5:0.95 and a 0.5% gain in mAP@0.5 over the baseline, alongside a reduction of 0.4 M parameters. Compared with v10Detect, GCCHead delivered superior performance in both detection precision and model complexity. Furthermore, the results indicate that GCCHead consistently maintained an advantage in detecting objects, demonstrating enhanced robustness in autonomous driving scenarios.

#### 4.5.4. Comparative Experiments Across Different Datasets

**Figure 10:**
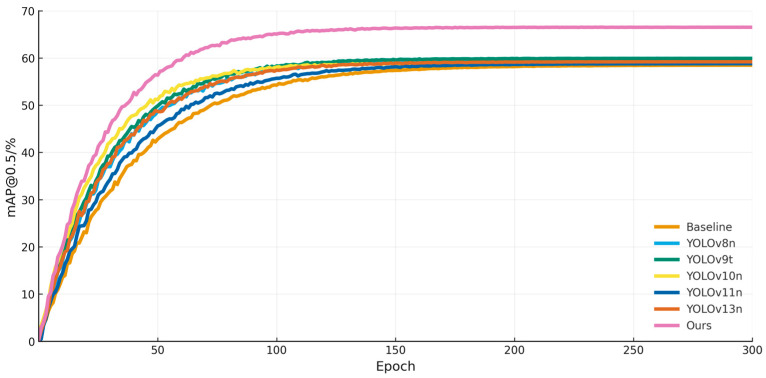
Training process of different models on SODA10M dataset.

RSO-YOLO was benchmarked against multiple lightweight detectors on the SODA10M dataset. The results, summarized in Table 6, show that methods such as YOLOv8n, YOLOv9t [34], YOLOv10n [35], YOLOv11n, and YOLOv13n [36] achieved higher accuracy than the baseline, but their overall gains remained limited. Moreover, these models exhibited varying degrees of inefficiency in computational metrics such as GFLOPs and FPS. For instance, YOLOv9t achieved 59.9% mAP@0.5, yet its computational complexity reached 7.9 GFLOPs, restricting deployment flexibility. Similarly, while YOLOv8n delivered higher detection accuracy, its parameter exceeded 3M, which imposes additional resource requirements. In contrast, the proposed RSO-YOLO achieved 66.5% mAP@0.5 and 46.6% mAP@0.5:0.95, outdoing all comparison methods. At the same time, RSO-YOLO maintained a lightweight design with only 2.2 M parameters and a computational complexity of 5.2 GFLOPs, thereby effectively controlling computational overhead. These results highlight that RSO-YOLO not only delivers substantial improvements in detection accuracy but also ensures efficiency, striking a favorable balance between performance and resource demands. Furthermore, its lightweight architecture underscores its strong potential for deployment. Figure 10 illustrates the proposed model’s consistent superiority over alternative methods on the SODA10M dataset, demonstrating its robust performance across the training process.

Comparative experiment on the BDD100K dataset was carried out to further evaluate the model’s generalization ability, with findings summarized in Table 7. Several mainstream lightweight detectors achieved overall accuracy comparable to the baseline but showed limited potential for improvement. In some cases, performance even declined, particularly on the mAP@0.5:0.95 metric. Moreover, these methods continued to face constraints in computational complexity and inference speed, making it difficult to attain a balance between efficiency and accuracy. In contrast, the proposed model achieved substantial gains of 10.7% and 3.9% over the baseline on mAP@0.5 and mAP@0.5:0.95 clearly outperforming all other methods. These findings confirm that the RSO-YOLO not only delivers high performance on the SODA10M dataset but also illustrates strong generalization capability and robust cross-dataset transferability. Taken together, the results indicate that the RSO-YOLO is well-adapted to deployment in diverse autonomous driving scenarios, where both reliability and efficiency are essential.

**Figure 11:**
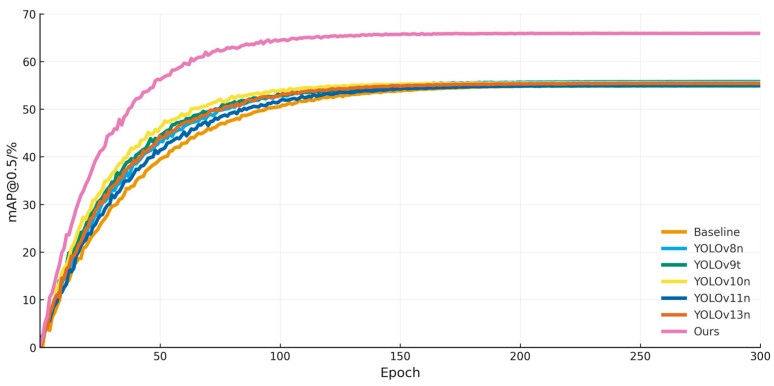
Training process of different models on BDD100K dataset.

Additionally, the model’s training process on the BDD100K dataset was documented, shown in Figure 11. Despite being applied to a dataset different from the one it was originally trained on, the improved model delivered consistently superior detection performance. Throughout the training process, its detection accuracy remained higher than that of all comparison models, thereby highlighting the model’s versatility, robustness, and strong adaptability to new domains.

**Figure 12:**
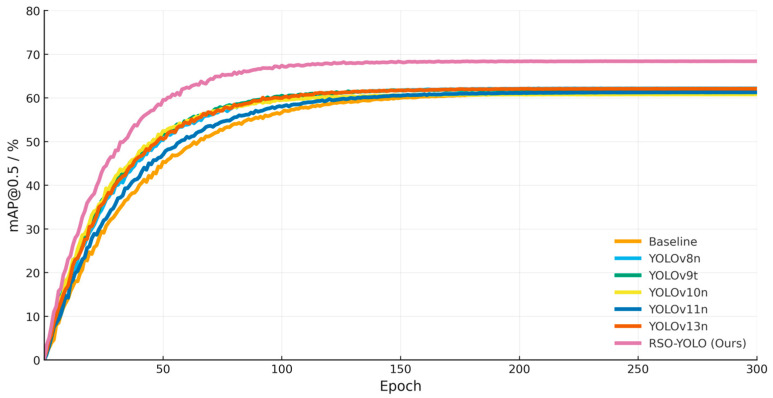
Training process of different models on FLIR ADAS dataset.

To further evaluate the generalization capability of the proposed model, experiments were conducted on the FLIR ADAS dataset, and the results are summarized in Table 8. The findings indicate that existing lightweight detectors exhibit relatively limited performance on this dataset. Although YOLOv8n and YOLOv13n achieve slightly higher baseline accuracy, they generally incur greater computational complexity. In contrast, the proposed RSO-YOLO demonstrates a substantial performance advantage, achieving mAP@0.5 and mAP@0.5:0.95 values of 68.4% and 35.4%, respectively, representing significant improvements over the baseline model. These results validate the effectiveness of the proposed architectural optimizations and underscore the model’s superior generalization capability. To analyze model performance, the training processes of different models were compared in terms of their convergence behavior on the FLIR ADAS dataset. As shown in Figure 12, all models exhibit stable convergence trends but differ in convergence speed and overall improvement. Throughout the entire training process, the proposed RSO-YOLO consistently maintains superior performance, converging faster and more stably, thereby demonstrating enhanced stability and generalization capability.

#### 4.5.5. Experimental Comparison with Other Improved Models

To further verify the model’s effectiveness, comparative experiments were conducted against advanced YOLO-based improvements proposed within the past two years on the SODA10M dataset. The experimental results shown in Table 9 show that Attention-YOLOv4 and RFCS-YOLO achieved mAP@0.5 values of 62.8% and 56.7%, respectively. However, their complex backbones, containing 65.7 M and 36.2 M parameters, made them unsuitable for lightweight and real-time deployment. Similarly, MEL-YOLO and YOLOv8-ghost-EMA reduced parameter counts but suffered from suboptimal accuracy, with mAP@0.5 values of only 53.8% and 55.6%. YOLO-BOS delivered stronger accuracy at 63.5% mAP@0.5, yet its parameter count exceeded 3M, limiting its efficiency.

In contrast, the RSO-YOLO reached a peak mAP@0.5 of 66.5%, outperforming all comparison models. With only 2.2 M parameters and an inference speed of 108 FPS, it significantly reduced model complexity while maintaining superior accuracy. Moreover, RSO-YOLO demonstrated exceptional stability in detecting small and occluded objects in complex scenes. By achieving lightweight processing without compromising detection performance, the model clearly demonstrates strong potential for real-world deployment in autonomous driving applications.

#### 4.5.6. Quantitative Experiments on Small and Occluded Object Detection

To further evaluate the detection performance of RSO-YOLO across different object scales and occlusion conditions, experiments were conducted on the small, medium, and large object subsets. As shown in Table 10, RSO-YOLO consistently outperforms the baseline model YOLOv12 across targets of all scales. The improvement is most pronounced for small objects, where the mAP@0.5 increases from 47.3% to 53.8%, indicating that the proposed architecture more effectively captures fine-grained feature information and enhances detection accuracy for small targets. For medium and large objects, RSO-YOLO also achieves steady performance gains, further confirming the model’s robustness across different scales.

Additionally, evaluations were conducted on the occluded and non-occluded subsets, with the corresponding results presented in Table 11. RSO-YOLO demonstrates superior detection performance under occlusion scenarios. For occluded objects, it improves mAP@0.5 and mAP@0.5:0.95 by 3.3% and 2.6%, respectively, while achieving even higher accuracy under non-occluded conditions. These results indicate that RSO-YOLO maintains strong object perception and discrimination capabilities in complex occlusion environments, thereby effectively enhancing overall detection performance.

### 4.6. Visualization Analysis

#### 4.6.1. Visualization of FECM Mechanism

**Figure 13:**
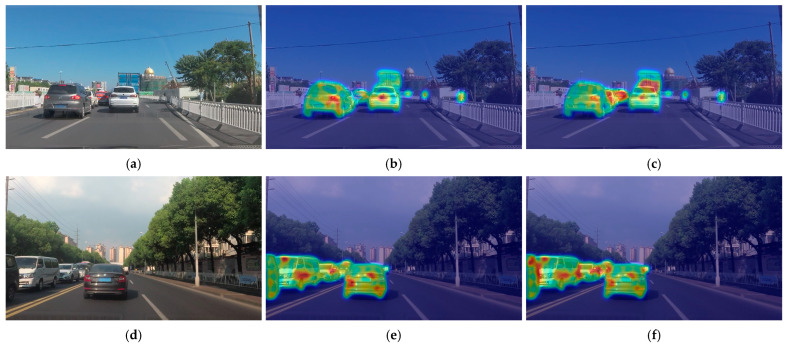
Heatmap comparison of detection results between YOLOv12 and YOLOv12 with the FECM: (**a**,**d**) are the original images; (**b**,**e**) are the detection results produced by YOLOv12; (**c**,**f**) are the detection results generated by YOLOv12 with the FECM. Colors represent attention intensity, where red and yellow indicate higher focus, and green indicates lower focus.

To verify the working mechanism of the FECM, heatmap visualizations were generated to compare YOLOv12 and YOLOv12 integrated with the FECM, as illustrated in Figure 13. Specifically, Figure 13a,d represent the original images; Figure 13b,e display the detection results of YOLOv12; and Figure 13c,f show the outputs of YOLOv12 with the FECM. As observed, Figure 13c,f exhibit more concentrated attention in the occluded regions compared with Figure 13b,e. These results demonstrate that the FECM effectively guides the model to extend its focus from visible areas to occluded regions, thereby compensating for semantic information loss and enhancing detection continuity and robustness under partial occlusion.

#### 4.6.2. Detection Results Visualization

**Figure 14:**
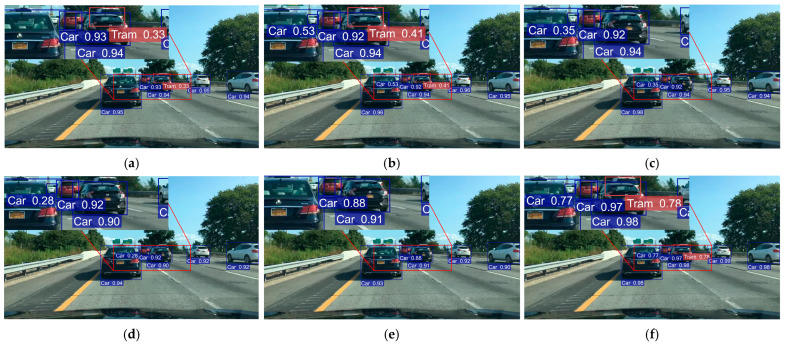
Detection results comparison under normal scenes: (**a**) YOLOv8n; (**b**) YOLOv9t; (**c**) YOLOv10n; (**d**) YOLOv11n; (**e**) YOLOv12n; (**f**) RSO-YOLO (Ours).

**Figure 15:**
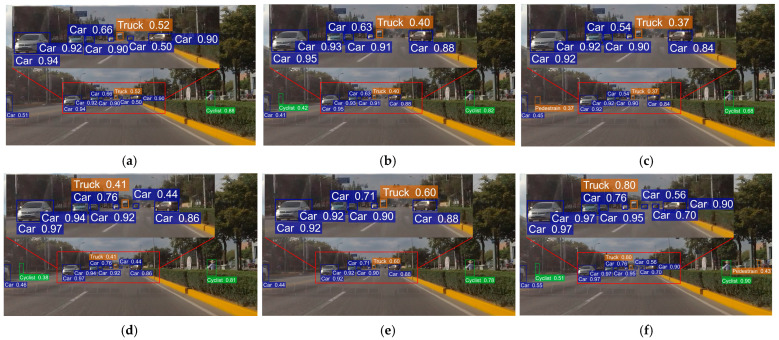
Detection results comparison under small target scenarios: (**a**) YOLOv8n; (**b**) YOLOv9t; (**c**) YOLOv10n; (**d**) YOLOv11n; (**e**) YOLOv12n; (**f**) RSO-YOLO (Ours).

**Figure 16:**
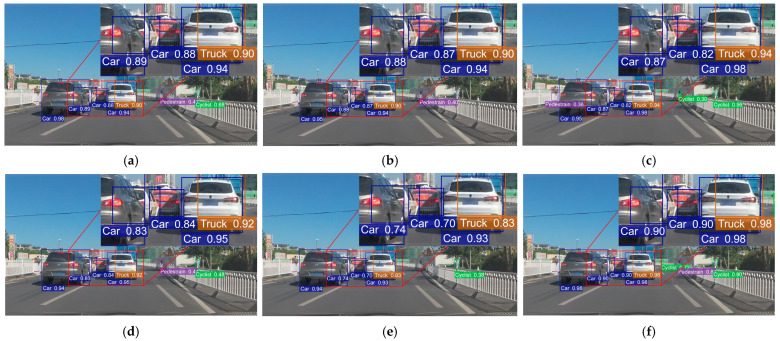
Detection results comparison under occlusion scenarios: (**a**) YOLOv8n; (**b**) YOLOv9t; (**c**) YOLOv10n; (**d**) YOLOv11n; (**e**) YOLOv12n; (**f**) RSO-YOLO (Ours).

To offer a clearer evaluation of algorithm performance, we conducted comparative experiments between (a) YOLOv8n, (b) YOLOv9t, (c) YOLOv10n, (d) YOLOv11n, (e) YOLOv12n and (f) RSO-YOLO. Three representative scenarios from the SODA10M dataset were selected for both training and visualization. Detection boxes were color-coded as follows: blue for Cars, red for Trams, yellow for Trucks, green for Cyclists, and purple for Pedestrians. The evaluation covered three representative scenarios: (1) A simple scene with few targets and minimal occlusion (Figure 14). (2) A scene containing small targets at long distances (Figure 15). (3) A complex scene with dense occlusions and strong background interference (Figure 16). The visual analysis results are shown in Figure 14, Figure 15 and Figure 16, which include zoomed-in views of key detection areas.

In the simple scene shown in Figure 14, all comparison models, YOLOv8n, YOLOv9t, YOLOv10n, YOLOv11n, and YOLOv12n, performed well in detecting objects. However, when minor occlusions occurred, YOLOv10n, YOLOv11n, and YOLOv12n produced missed detections. Although YOLOv8n and YOLOv9t detected partially occluded objects, their detection accuracy was notably lower than that of RSO-YOLO. In contrast, RSO-YOLO effectively suppressed background interference and achieved a marked improvement in detection accuracy.

In the distant small-object and complex-occlusion scenarios shown in Figure 15 and Figure 16, all comparison models exhibited both FP and FN. For instance, in long-range small-object detection, all models except RSO-YOLO failed to detect Pedestrians, while YOLOv8n, YOLOv10n, and YOLOv12n also failed to detect Cyclists. Moreover, YOLOv10n misclassified a Cyclist as a Pedestrian due to obstruction by roadside barriers. Similarly, in complex-occlusion scenes, YOLOv10n misclassified potted plants along the roadside as Pedestrians. By contrast, RSO-YOLO maintained stable detection across these challenging conditions, effectively reducing both FN and FP.

In summary, RSO-YOLO demonstrated superior robustness and detection accuracy across scenarios of varying complexity, validating its potential for deployment in autonomous driving.

## 5. Conclusions

To overcome the constraints of current models in effectively managing small targets, occlusion issues, and lightweight demands, this study proposed an innovative model, RSO-YOLO. Methodologically, the original neck network was replaced with BiFPN and SPD-Conv, enabling efficient multi-scale feature fusion while preserving fine-grained information. This design improved both computational efficiency and detection performance. Additionally, a P2 detection head was introduced, extending the network to a four-head structure and further enhancing detection capability for small objects. To tackle occlusion, a novel FECM was proposed. FECM leveraged multi-scale branch-cooperative channels and spatial attention to amplify effective features in unoccluded regions. High-confidence cues extracted from these regions were then transferred to occluded areas, effectively compensating for missing information and significantly improving robustness under occlusion. Finally, a lightweight GCCHead detection head was developed, incorporating the GCCM designed in this study. By grouping features and adaptively boosting responses in key regions, GCCM enhanced detection accuracy while simultaneously reducing parameters and computational complexity, thereby striking an effective balance between accuracy and real-time inference speed. Experimental results demonstrated that RSO-YOLO achieved an 8.0% improvement in mAP@0.5 on the SODA10M dataset, a 10.7% improvement on the BDD100K dataset, and a 7.2% improvement on the FLIR ADAS dataset, outperforming multiple mainstream detection models. Moreover, quantitative analysis confirmed that RSO-YOLO significantly improved detection accuracy for small objects and for occluded targets. These results comprehensively verify the model’s strong adaptability and high reliability across diverse autonomous driving scenarios. Furthermore, visual analysis on SODA10M confirmed the model’s performance in detecting occluded and small targets, underscoring its strong potential for deployment in real-world autonomous driving applications.

Although RSO-YOLO achieved significant improvements on both the SODA10M and BDD100K datasets, challenges remain in scenarios involving extreme occlusion, nighttime conditions, and adverse weather, as well as in its cross-domain generalization capability. Future work will focus on enhancing generalization and robustness in complex environments through strategies such as data augmentation and multimodal fusion. These improvements are expected to further strengthen the model’s performance for autonomous-driving detection tasks under diverse and challenging conditions.

## Figures and Tables

**Table 1:** Ablation experimental results on the SODA10M dataset.

Model	BiFPN	P2	GCCHead	FECM	P (%)	R (%)	mAP@0.5 (%)	mAP@0.5:0.95 (%)	Params (M)	FPS	GFLOPs
Baseline					69.4	51.6	58.5	39.6	2.6	120	6.5
A	√				72.7	53.0	59.2	40.2	2.2	130	4.2
B		√			68.1	57.0	63.0	44.6	2.7	117	8.4
C			√		72.6	52.6	59.0	40.0	2.2	116	4.9
D				√	67.7	53.7	59.3	40.4	2.6	100	6.6
AB	√	√			68.6	59.1	64.5	44.7	2.4	118	5.9
ABC	√	√	√		69.9	60.5	65.5	45.6	2.1	110	4.8
ABD	√	√		√	72.8	57.8	65.4	45.5	2.6	104	6.5
ABCD (Ours)	√	√	√	√	77.1	57.6	66.5	46.6	2.2	108	5.2

**Table 2:** Ablation experimental results on the BDD100K dataset.

Model	BiFPN	P2	GCCHead	FECM	P (%)	R (%)	mAP@0.5 (%)	mAP@0.5:0.95 (%)	Params (M)	FPS	GFLOPs
Baseline					65.2	48.3	55.2	27.4	2.6	118	6.5
A	√				68.5	49.8	56.0	28.1	2.2	128	4.2
B		√			64.0	53.5	59.8	29.2	2.7	105	8.4
C			√		67.3	49.6	55.9	27.9	2.2	114	4.9
D				√	63.8	50.4	56.5	28.3	2.6	104	6.6
AB	√	√			64.5	55.6	61.3	30.5	2.4	108	5.9
ABC	√	√	√		65.8	54.1	62.2	30.9	2.1	116	4.8
ABD	√	√		√	68.6	54.3	62.4	31.1	2.6	102	6.5
ABCD	√	√	√	√	72.5	56.8	65.9	31.3	2.2	106	5.2

**Table 3:** Results of Neck Network Comparison Experiment.

Model	mAP@0.5 (%)	mAP@0.5:0.95 (%)	Params (M)
Baseline	58.5	39.6	2.6
GFPN	59.6	40.6	3.0
BiFPN (ours)	59.2	40.2	2.2

**Table 4:** Performance Comparison of Occlusion Object Detection Based on FECM.

Model	FECM	mAP@0.5 (%)	mAP@0.5:0.95 (%)	Params (M)
Baseline		46.0	27.3	2.6
	√	47.2	28.2	2.6

**Table 5:** Results of Detection Head Comparison Experiment.

Model	mAP@0.5 (%)	mAP@0.5:0.95 (%)	Params (M)
Baseline	58.5	39.6	2.6
v10Detect	58.0	38.6	2.9
GCCHead (ours)	59.0	40.0	2.2

**Table 6:** Comparative experiments of different models on SODA10M dataset.

Model	mAP@0.5 (%)	mAP@0.5:0.95 (%)	Params (M)	FPS	GFLOPs
Baseline	58.5	39.6	2.6	120	6.5
YOLOv8n	59.6	40.4	3.2	103	8.7
YOLOv9t	59.9	40.6	2.0	94	7.9
YOLOv10n	58.9	39.9	2.3	182	6.7
YOLOv11n	58.8	39.9	2.6	169	6.5
YOLOv13n	59.2	40.1	2.4	150	6.4
RSO-YOLO (Ours)	66.5	46.6	2.2	108	5.2

**Table 7:** Comparative experiments of different models on BDD100K dataset.

Model	mAP@0.5 (%)	mAP@0.5:0.95 (%)	Params (M)	FPS	GFLOPs
Baseline	55.2	27.4	2.6	120	6.5
YOLOv8n	55.8	27.8	3.2	222	8.7
YOLOv9t	54.9	26.7	2.0	96	7.9
YOLOv10n	55.5	27.6	2.3	137	6.7
YOLOv11n	55.1	26.9	2.6	151	6.5
YOLOv13n	55.4	27.4	2.4	140	6.4
RSO-YOLO (Ours)	65.9	31.3	2.2	108	5.2

**Table 8:** Comparative experiments of different models on FLIR ADAS dataset.

Model	mAP@0.5 (%)	mAP@0.5:0.95 (%)	Params (M)	FPS	GFLOPs
Baseline	61.2	29.2	2.6	116	6.5
YOLOv8n	62.2	30.1	3.2	125	8.7
YOLOv9t	62.0	29.6	2.0	101	7.9
YOLOv10n	60.8	29.0	2.3	121	6.7
YOLOv11n	61.3	29.3	2.6	132	6.5
YOLOv13n	62.1	29.8	2.4	130	6.4
RSO-YOLO (Ours)	68.4	35.4	2.2	126	5.2

**Table 9:** Comparison of other improved models.

Model	Backbone	mAP@0.5 (%)	Params (M)	FPS
Attention-YOLOv4	YOLOv4	62.8	65.7	42
MEL-YOLO	YOLOv5	53.8	3.1	46.2
YOLOv8-ghost-EMA	YOLOv8	55.6	2.6	106
RFCS-YOLO	YOLOv7	56.7	36.2	68
YOLO-BOS	YOLOv8	63.5	3.27	100
RSO-YOLO (Ours)	YOLOv12	66.5	2.2	108

**Table 10:** Comparison of Detection Performance under Different Object Scales.

Category	mAP@0.5 (%)		mAP@0.5:0.95 (%)	
	YOLOv12	RSO-YOLO	YOLOv12	RSO-YOLO
Small object subset	47.3	53.8	26.8	32.5
Medium object subset	60.4	63.4	41.2	43.8
Large object subset	67.5	69.0	52.3	54.2
Overall	58.5	66.5	39.6	46.6

**Table 11:** Comparison of Detection Performance under Occlusion Conditions.

Category	mAP@0.5 (%)		mAP@0.5:0.95 (%)	
	YOLOv12	RSO-YOLO	YOLOv12	RSO-YOLO
Occluded object subset	46.0	49.3	25.8	28.4
Non-occluded object subset	62.6	70.2	44.2	50.4
Overall	58.5	66.5	39.6	46.6

## Data Availability

Data related to the current study are available from the corresponding author upon reasonable request. The code used during the study is available from the corresponding author upon request.
